# Cerebral Venous Sinus Thrombosis in Pediatric Critical Care

**DOI:** 10.1097/CCE.0000000000001418

**Published:** 2026-05-25

**Authors:** Christina Maratta, Melany Gaetani, Nicole K. McKinnon, David Anthony, Sahrish Masood, Elizabeth Pulcine, Haifa Mtaweh

**Affiliations:** 1 Division of Pediatric Critical Care, McGill University Health Centre, Montreal, QC, Canada.; 2 Department of Pediatrics, McGill University, Montreal, QC, Canada.; 3 Department of Critical Care Medicine, Hospital for Sick Children, Toronto, ON, Canada.; 4 Department of Pediatrics, University of Toronto, Toronto, ON, Canada.; 5 Interdepartmental Division of Critical Care Medicine, University of Toronto, Toronto, ON, Canada.; 6 Child Health Evaluative Sciences, SickKids Research Institute, Toronto, ON, Canada.; 7 Division of Neurosciences and Mental Health, SickKids Research Institute, Toronto, ON, Canada.; 8 Department of Information Services, Information Services, Hospital for Sick Children, Toronto, ON, Canada.; 9 Division of Neurology, Department of Pediatrics, University of Toronto, Toronto, ON, Canada.

**Keywords:** cerebral venous sinus thrombosis, outcomes, pediatrics, stroke

## Abstract

**IMPORTANCE:**

Pediatric cerebral venous sinus thrombosis (CVST) is being increasingly recognized and can pose substantial risks of morbidity and mortality. Data on the epidemiology, management, and outcomes of CVST in the PICU remain limited.

**OBJECTIVES:**

To describe the clinical characteristics, management, and outcomes of critically ill children with CVST during their admission to the PICU.

**DESIGN, SETTING, AND PARTICIPANTS:**

We conducted a retrospective observational cohort study in a quaternary PICU in Toronto, Canada, between 2018 and 2023. Patients 18 years old and younger with acute primary CVST (CVST being the primary indication for ICU admission) and secondary CVST (diagnosis during an admission for an alternative diagnosis) were included in this study.

**MAIN OUTCOMES AND MEASURES:**

The primary outcome was in-hospital mortality. Descriptive statistics were used to describe characteristics and outcomes.

**RESULTS:**

Thirty patients were admitted with a diagnosis of CVST: 19 (63%) primary, 11 (37%) secondary. Fourteen (47%) had an associated cerebral infarct, and nine (30%) had an associated intracranial hemorrhage. The most common condition associated with secondary CVST was a brain disease requiring neurosurgical intervention (5/11). Five (17%) children with CVST died in this study, of which four had a primary CVST. Children residing in neighborhoods with increased marginalization were disproportionally represented in this cohort.

**CONCLUSIONS AND RELEVANCE:**

Primary CVST is more common than secondary and is associated with significant mortality. The disproportionate impact on marginalized children emphasizes the need for heightened awareness and determination of factors associated with this finding.

**KEY POINTS**

**Question**: What are the clinical characteristics, management patterns, and outcomes of critically ill children with primary and secondary cerebral venous sinus thrombosis (CVST) admitted to a quaternary PICU?

**Findings**: In 30 children, primary CVST predominated (63%) and was associated with longer symptom duration and higher mortality (17% overall). Nearly one-half had cerebral infarcts and 30% intracranial hemorrhage, with frequent need for intensive interventions. Secondary CVST was often linked to neurosurgical conditions. Children from marginalized neighborhoods were overrepresented.

**Meaning**: Pediatric CVST in critical illness carries substantial morbidity and mortality, particularly for primary cases, and reveals important social inequities requiring targeted investigation and intervention.

Cerebral venous sinus thrombosis (CVST) is formation of a thrombus in the superficial and/or deep venous sinuses that can be nonocclusive or fully occlusive leading to a secondary venous infarction (1). Due to inadequate intracranial venous drainage, clinically significant CVSTs can result in raised intracranial pressure (ICP), cerebral edema, nonhemorrhagic, or hemorrhagic venous infarction, secondary seizures and, if left untreated, even death. Risk factors for CVST in children fall into one of four main categories, but are usually multifactorial: 1) infections of the head and neck, 2) chronic systemic illness and their concomitant treatments leading to hypercoagulability (liver disease, nephrotic syndrome, systemic lupus erythematosus, malignancy, iron-deficiency anemia, sepsis), 3) inherited thrombophilia, and 4) severe dehydration (1–6). Idiopathic CVST is rare in children. Both the incident and national burden of pediatric CVST have increased in the United States over the past 15 years (7). Reported disease incidence range from 0.6 to 1.1/100,000 children per year, as well as an increase in age- and sex-standardized CVST incidence of 3.8% annually, and an overall annual increase of CVST admissions of 4.9% (7, 8).

There are no clinical markers or specific neurologic signs and symptoms of CVST, and diagnosis relies primarily on a high index of suspicion. Accordingly, CVST varies in the clinical presentation based on age. Neonates and infants usually present with seizures and irritability, vs. children and adolescents presenting with headache and signs of high ICP. Clinical presentation severity can vary significantly, depending on the underlying disease, the specific venous sinus involved, the extent to which collateral venous drainage has formed, and the influence of medical or surgical management approaches (3, 6, 9). Underlying patient factors such as socioeconomic status could have a role in disease modification, similar to its contribution to nutritional adequacy and anemia (10), neurocognitive (11), and clinical outcomes (12). Finally, CVST carries a significant risk for morbidity and mortality, with reported mortality ranges of 3–12% and neurologic disability affecting up to 62% of survivors (9, 13, 14).

Making the diagnosis of CVST requires a high index of suspicion, particularly in the critical care setting where the illness increases the risk of thromboembolism and the neurologic assessment is confounded by sedatives, use of mechanical ventilation, and neuromuscular blockade. Currently, limited data exist on the epidemiology and management of CVST in the pediatric critical care setting. Improved description of the presentation, diagnosis, and management of CVST in critically ill children could aid in early recognition and prognostication for this unique population.

This study aimed to describe the clinical characteristics, management strategies, and outcomes of critically ill children with primary and secondary CVST during their admission to a single-center quaternary ICU setting.

## METHODS

### Study Design and Setting

We performed a retrospective cohort study in a combined 44-bed Pediatric and Cardiac Critical Care Unit at the Hospital for Sick Children (SickKids) in Toronto, Ontario, Canada. Eligible patients were children 0–18 years old who had a diagnosis of CVST during their ICU admission between June 1, 2018, and June 30, 2023. Patients were identified using the *International Classification of Diseases*, 10th Revision codes: G08, i63.6, i67.6 then by screening all primary and concomitant diagnoses associated with the ICU admission from the electronic medical chart. Charts and radiologic reports were then reviewed to confirm the diagnosis of the CVST. Institutional practice includes a multidisciplinary review between ICU, thrombosis, neurology (acute care/stroke team), neurosurgery, and radiology to determine the most suitable clinical care practices for the patient.

The study procedures were followed in accordance with the ethical standards of the Helsinki Declaration of 1975 and were approved by the institutional research ethics board at SickKids (number: 1000080975, Title: Cerebral sinus venous thromboses in pediatric critical care: a retrospective cohort study, Initial approval: August 30, 2023). The reporting is in accordance with the Strengthening the Reporting of Observational Studies in Epidemiology guidelines (15).

### Outcomes

The primary outcome was hospital mortality. Secondary outcomes included procedural therapies, length of stay, and function at discharge.

### Data Abstraction and Management

We abstracted demographic and clinical characteristics from the electronic medical records and by manual chart review. Prior medical history, history of presenting illness as reported in the admission note, vital signs, initial laboratory values, therapies received in ICU, diagnostic imaging reports, and electroencephalogram reports were reviewed and recorded. Sinus thromboses locations on presentation were recorded and classified as superficial or deep. Deep sinus thromboses included those present in the internal cerebral veins, inferior sagittal sinus, vein of Galen, and straight sinus (including torcula). Superficial sinus thromboses included those present in the superior sagittal sinus, transverse sinuses, sigmoid sinuses, internal jugular bulbs/veins, and the cortical veins (16, 17). Medication administrations including antiepileptic medications, anticoagulation and hyperosmolar therapies were recorded throughout the ICU admission. Anticoagulation medication includes heparin to achieve effective anticoagulation determined by antiXa levels and guided by thrombosis team, after which transition to enoxaparin occurs at discretion of critical care and thrombosis teams. Hyperosmolar therapy includes 3% saline and mannitol, the only two available products at our institution. Interventional procedures including thrombectomy, insertion of an ICP monitor, and decompressive craniectomy were abstracted by reviewing procedure and operative code and notes. Therapy initiation and duration days were defined by calendar days, where day 1 was the first day of the ICU admission. Functional Status Scale (FSS), Pediatric Cerebral Performance Category (PCPC), and Pediatric Overall Performance Category (POPC) were calculated at baseline and at hospital discharge by performing a chart review (18, 19). PCPC or POPC scores of 6 represent death. Baseline functional scores were ascertained from caregiver history and reflective of preadmission function. Discharge summaries and death summaries were reviewed to determine survival, as well as to describe the occurrence of withdrawal of life-sustaining therapies or organ donation after death. The 2021 version of the Ontario Marginalization Index (ON-Marg) was used for patient proxy of poverty (Material Resources dimension). The ON-Marg Racialized and Newcomer Population, which uses the self-reported census variables “visible minority” and “recent immigration,” was used as a proxy for experience of race/ethnicity. ON-Marg is an area-based index of marginalization that uses Canadian census data to approximate dimensions of marginalization, and for which quintiles are ascribed to an individual based on their postal code. Quintile 5 indicates that the individual resides in one of the 20% most marginalized areas in Ontario (20).

Primary CVST was defined as an acute diagnosis of CVST resulting in critical illness and being the primary indication for ICU admission. Secondary CVST was defined as the diagnosis of CVST during an admission for an alternative diagnosis (disease). The possible causes or risk factors for secondary CVST that were collected included: brain infection, bleed, stroke, anemia, dehydration, prothrombotic disorders, liver disease, renal disease, rheumatologic conditions, metabolic disorders, neurosurgical procedures, and platelet abnormalities.

### Statistical Analysis

Demographic and clinical characteristics, as well as outcomes, are described using frequencies and percentages for categorical variables, means and sds for normally distributed continuous variables, and medians and interquartile ranges for nonparametric continuous variables. The primary outcome was measured as the proportion of ICU patients with CVST who died during the incident hospitalization. Chi-square or Fisher exact test was used to compare categorical data and Wilcoxon rank sum test for the comparison of medians. Missing data were not imputed. Analysis was performed with R, Version 4.1.0 (The R Foundation for Statistical Computing, Vienna, Austria).

## RESULTS

### Cohort Description

A total of 30 patients were admitted to the ICU at SickKids between June 1, 2018 and June 30, 2023, with a diagnosis of CVST. CT with contrast or venogram was used to obtain the diagnosis in 23 (76%) patients and MRI with venography in 7 (24%) patients. The median (Q1, Q3) age was 6 years (1.3, 13.3) and 17 (57%) were of male sex (**Table 1**). Symptoms were present for a median of 3 days before presentation. Fatigue or lethargy was the predominant symptom in 19 of 30 patients (63%) followed by seizures in 17 patients (57%). A similar distribution of generalized and focal seizures was observed in those 17 patients. Median admission Glasgow Coma Scale (GCS) score was 10 (3, 15), and 60% of patients demonstrated anemia (hemoglobin < 110 g/L at admission) (**Supplemental Table 1**, https://links.lww.com/CCX/B630).

**TABLE 1.** Patient Characteristics and Presenting Features

Characteristic	All (*n* = 30)	Primary Cerebral Venous Sinus Thrombosis (*n* = 19)	Secondary Cerebral Venous Sinus Thrombosis (*n* = 11)
Age (mo), median (IQR)	72 (15,159)	67 (23, 156)	79 (2, 164)
Sex, *n* (%)			
Male	17 (57)	10 (53)	7 (64)
Female	13 (43)	9 (47)	4 (36)
Days of symptoms before presentation	3 (1, 5)	4 (1.5, 7)	1 (0, 2.5)
Presenting symptoms^a^			
Dehydration	14 (47)	12 (63)	2 (18)
Headache	12 (40)	10 (53)	2 (18)
Altered level of consciousness	8 (27)	6 (32)	2 (18)
Seizures, any	17 (57)	10 (53)	7 (64)
Generalized seizures	8/17 (47)	5/10 (50)	3/7 (43)
Focal seizures	7/17 (41)	4/10 (40)	3/7 (43)
Fatigue, lethargy, weakness	19 (63)	14 (74)	5 (45)
Focal motor neurologic deficit	8 (27)	5 (26)	3 (27)
Cranial nerve palsy	3 (10)	1 (5)	2 (18)
Otitis	2 (7)	2 (11)	0
Fever	11 (37)	6 (32)	5 (45)
History of vomiting	14 (47)	9 (47)	5 (45)
Admission diagnosis			
Arteriovenous malformation	1 (3)	0	1 (9)
Cerebral sinus venous thrombosis	15 (50)	15 (79)	0
Hypovolemic shock	1 (3)	0	1 (9)
Intracranial hemorrhage	1 (3)	1 (5)	0
Intracranial lesion	1 (3)	0	1 (9)
Meningitis	1 (3)	0	1 (9)
Metabolic crisis	1 (3)	0	1 (9)
Persistent pulmonary hypertension of the newborn	1 (3)	0	1 (9)
Seizure	3 (10)	1 (5)	2 (18)
Sickle cell disease	1 (3)	1 (5)	0
Thrombocytopenia	1 (3)	1 (5)	0
Trauma	3 (10)	0	3 (27)
Admission Glasgow Coma Scale, median (IQR)	10 (3.3, 15)	14 (7, 15)	6 (3, 10)

IQR = interquartile range.

a Additional presenting symptoms of interest that no patients presented with included respiratory arrest, apnea, and pharyngitis.

**Figure 1.**
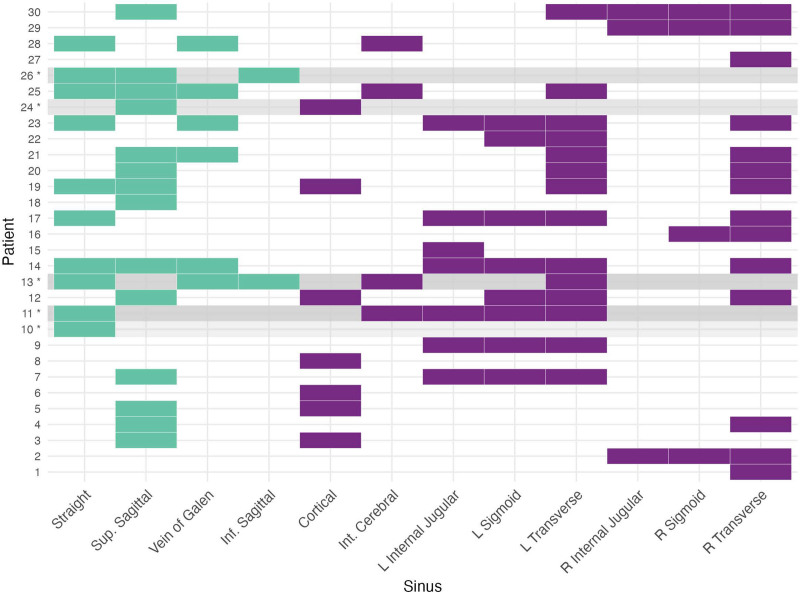
Distribution of venous thromboses. Two of thirty patients had only occlusive deep sinus thromboses, nine had occlusive thrombi in the superficial and deep systems, and the remaining 19 patients had superficial venous sinus thromboses. * and *gray* background represent patients who died, *purple* background denotes superficial venous system, *teal* background denotes deep venous system. Inf = inferior, Int = internal, L = left, R = right, Sup = superior.

Two patients had a diagnosis of meningitis, and one of diabetic ketoacidosis (Table 1), six patients (20%) had undergone neurosurgical intervention (three craniotomies for bleed, arteriovenous malformation resection, craniosynostosis, one decompressive craniotomy, one hemispherectomy, one ventricular drain). Two patients (7%) had suffered traumatic brain injury, one diagnosed with CVST at time of presentation and the second one day later, and 2 patients (7%) had suffered abusive head trauma. There was a median of 3 days (1, 5) of symptoms before presentation. Two of 30 patients (6%) had only occlusive deep sinus venous thromboses, 9 of 30 (30%) had occlusive thrombi in both the superficial and deep systems, and the remaining 19 patients (63%) had superficial venous sinus thromboses (three were of nonocclusive nature) (**Fig. 1**).

### Hospital Course and Outcomes—Full Cohort

**Figure 2.**
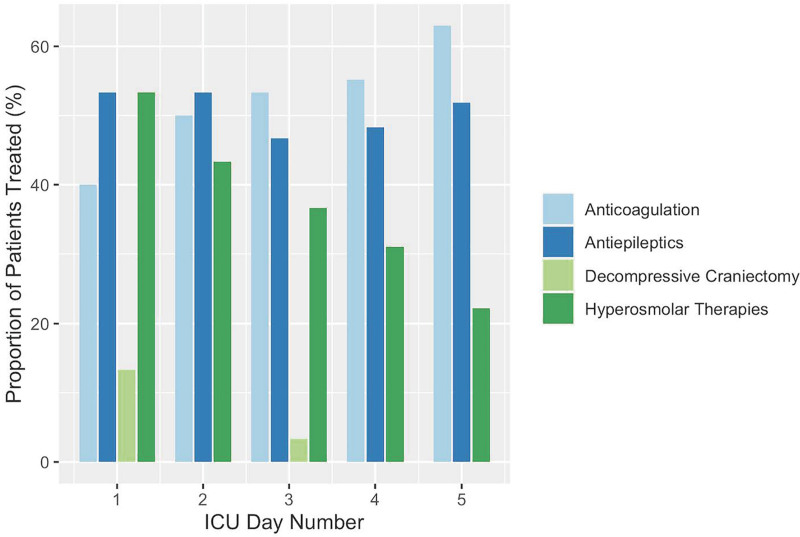
Therapies received by ICU day. Systemic anticoagulation and antiepileptics were used in approximately 50% of patients. Decompressive craniectomy occurred early in ICU admission and the use of hyperosmolar therapy decreased as the number of ICU admission days increased.

Among the 30 patients with CVST, 16 (53%) were mechanically ventilated, 1 patient had an ICP monitor placed secondary to the presence of the CVST, and 5 patients (26%) underwent decompressive craniectomy for CVST-related complications. No patients underwent endovascular therapy, and a single patient had systemic tissue-type plasminogen activator (TPA) therapy (**Table 2**). The single patient who received systemic TPA also had a pulmonary embolus and multiple deep vein thromboses. Decompressive craniectomy occurred early in ICU admission and the use of hyperosmolar therapy decreased as the number of ICU admission days increased. Systemic anticoagulation was used in 24 patients (80%) during the ICU stay, heparin alone in 4 patients, and heparin followed by enoxaparin in 20 patients. Five of the patients who were not on anticoagulation had an intracranial hemorrhage and one had an underlying genetic condition with diffuse thromboses and cerebral edema who underwent withdrawal of life-sustaining therapy. Antiepileptics were used in approximately 50% of patients (**Fig. 2**).

**TABLE 2.** Complications, Interventions, and Outcomes

Primary and Secondary Cerebral Venous Sinus Thrombosis Complications, Interventions, Outcomes	All (*n* = 30)	Alive (*n* = 25)	Dead (*n* = 5)	*p*
Primary CVST	*n* = 19	*n* = 15	*n* = 4	
CVST complications				
CVST-associated infarct	11 (58)	8 (53)	3 (75)	0.6
CVST-associated intracranial hemorrhage	7 (37)	4 (27)	3 (75)	0.12
Interventions or therapies^a^				
Invasive mechanical ventilation	8 (42)	4 (27)	4 (100)	0.018
ICP monitor in place, any	1 (5)	1 (7)	0	> 0.9
ICP monitor insertion for CVST	1 (5)	1 (7)	0	
Decompressive craniectomy	5 (26)	3 (20)	2 (50)	0.3
Clinical outcomes				
Hospital LOS in hours, median (IQR)	321 (141, 409)	377 (179, 431)	96 (76, 135)	0.009
No CPR order	4 (21)	0	4 (100)	< 0.001
Withdrawal of life-sustaining therapy	3 (16)	0	3 (75)	0.004
Donation after death by circulatory criteria	3 (16)	0	3 (75)	0.004
Secondary CVST	*n = 11*	*n = 10*	*n = 1*	
CVST complications				
CVST-associated infarct	3 (27)	3 (30)	0	> 0.9
CVST-associated intracranial hemorrhage	2 (18)	2 (20)	0	> 0.9
Interventions or therapies^b^				
Invasive mechanical ventilation	8 (73)	8 (80)	9	0.3
ICP monitor in place, any	2 (18)	2 (20)	0	> 0.9
External ventricular drain in place, any	4 (36)	3 (30)	1 (100)	0.4
Systemic tissue plasminogen activator	1 (9)	1 (1)	0	> 0.9
Clinical outcomes^c^				
Hospital LOS in hours, median (IQR)	759 (463, 1333)	1004 (557, 1360)	113	0.2
No CPR order	2 (18)	1 (10)	1 (100)	0.2

CPR = cardiopulmonary resuscitation, CVST = cerebral venous sinus thrombosis, ICP = intracranial pressure, IQR = interquartile range, LOS = length of stay, WLST = withdrawal of life-sustaining therapy.

a None of the patients had an external ventricular drain (EVD) inserted for any cause, or EVD for cerebral venous sinus thrombosis (CVST), or endovascular therapy, or systemic tissue plasminogen activator.

b None of the patients had an ICP monitor insertion for CVST, or EVD insertion for CVST, or decompressive craniectomy, or endovascular therapy.

c None of the patients had withdrawal of life-sustaining therapy or donation after Determination by Circulatory Criteria.

*p* values were generated using Chi-square or Fisher exact tests for proportions, and Wilcoxon rank sum tests for comparisons of medians.

Five of the 30 children (17%) with CVST in this study died (Table 2). Only one of five patients who died had superficial venous system thromboses, whereas the remaining four had either deep or combined thromboses. One of the five had a recent neurosurgical intervention, two had anemia at admission and three had CVST-associated intracranial hemorrhage. Three of the patients who underwent decompressive craniectomy survived to hospital discharge (Table 2).

### Differentiating Primary and Secondary CVST: History and Presentation

Acute primary CVST was present in 19 of the 30 cases (63%) and secondary CVST in 11 of 30 (37%). Patients with primary CVST had a longer reported duration of symptoms with a median of 4 days (1.5, 7) in comparison to 1 day (0, 2.5) for those with secondary CVST. Stratified by primary or secondary CVST, the presenting symptoms varied. In those with primary CVST the presenting symptoms were predominantly fatigue or lethargy in 14 of 19 (74%), followed by dehydration in 12 of 19 (63%). The predominant symptom in those with secondary CVST was seizures in 7 of 11 (64%), followed by fatigue, fever, or history of vomiting in 5 of 11 (45%) (Table 1). GCS at admission was lower for patients with secondary CVST with a median score of 6 (3, 10) in comparison to those with primary CVST presenting with a GCS of 14 (7, 15). Anemia was present in 13 (68%) of patients with primary and 5 (45%) in those with secondary CVST. A preceding history of anemia was found more commonly in those with primary CVST (median [interquartile range] hemoglobin 102 (75, 115) gr/L), whereas a preceding neurosurgical intervention was more common in those with secondary CVST 5 (45%).

A disproportionate number of children in this cohort (19, 69%) were determined to be living in neighborhoods with the greatest material deprivation (Quintiles 4 or 5) (Supplemental Table 1, https://links.lww.com/CCX/B630). Furthermore, 50% of patients with both primary and secondary CVST were in the fifth ON-Marg Racialized and Newcomer Population quintile, indicative of disproportionate marginalization within this dimension. Across both Material Resource quintiles and Racialized and Newcomer Population quintiles, there were no significant differences in days of symptoms before presentation, number of visits before admission, presenting symptoms, history of either anemia, or iron-deficiency anemia, nor any differences across quintiles in laboratory diagnosis of anemia at presentation.

### Differentiating Primary and Secondary CVST: Hospital Course and Outcomes

Nearly two-thirds of children with primary CVST had a cerebral infarct associated with the CVST and one-third had an associated intracranial hemorrhage. In those with secondary CVST only approximately one-third developed an associated infarct and one-fifth developed an associated hemorrhage. The median hospital length of stay was 13.4 days (5.9, 17) for those with primary CVST vs. 31.6 days (19.3, 55.5) for those with secondary CVST (Table 2).

**Figure 3.**
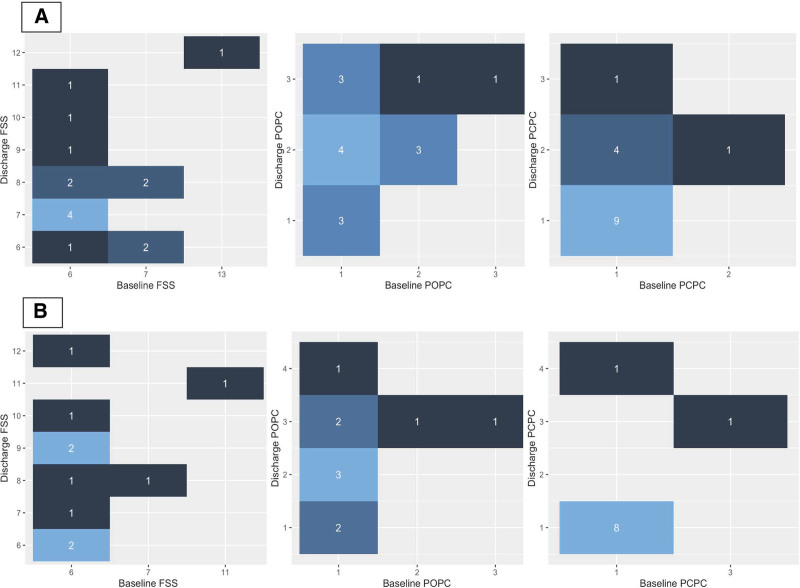
Patient functional outcomes after cerebral venous sinus thrombosis (CVST). **A**, Patients with primary CVST who survived to hospital discharge (*n* = 15). **B**, Patients with secondary CVST who survived to hospital discharge (*n* = 10). The number of patients in each category both at baseline and at discharge are depicted in *white* numbers on the figure. The lighter the *hue of blue*, the greater the number of patients in that category. Patients who did not survive are not included in this figure. FSS = Functional Status Scale, POPC = Pediatric Overall Performance Category, PCPC = Pediatric Cognitive Performance Category.

Four of 19 children (21%) with primary CVST died compared with 1 patient (10%) of those with secondary CVST (Table 2). Of the four children with primary CVST who died, 75% had both associated infarct and/or hemorrhage, two underwent decompressive craniectomy, all had a do-not-resuscitate order in their chart, and 75% proceeded with organ donation after death determination by circulatory criteria. Among those with primary CVST who survived, five of 15 (33%) had a change in their cognitive function scores (PCPC) and 8 (53%) had a change in their overall function scores (POPC) at hospital discharge. Of those with secondary CVST, 1 of 10 (10%) had a change in their PCPC, and 7 of 10 (70%) had a change in their POPC scores (**Fig. 3**). Children with primary and secondary CVST had median (Q1, Q3) FSS of 6 (6, 6.5), with 7 patients with secondary CVST and 11 with primary CVST having an increase (worse) of at least 1 in their FSS (Supplemental Table 1, https://links.lww.com/CCX/B630 and Fig. 3). There were however, no statistically significant differences in any functional outcome scores, hospital length of stay, or mortality.

## DISCUSSION

This study describes the course of children admitted to a PICU with cerebral venous sinus thromboses over a 5-year period. We report four main findings. First, primary CVST is a more common finding than secondary CVST outside the neonatal period in children who require ICU admission. Second, patients with primary CVST in this cohort reported having symptoms for longer periods before presenting to the ICU and had significant associated intracranial complications. Third, the predominant symptom for the presentation of secondary CVST is seizures (64%), compared with the more nonspecific lethargy and headaches commonly reported in primary CVST. Finally, we have identified that children residing in neighborhoods with increased marginalization are disproportionally represented in this cohort of patients.

CVST has primarily been described as a secondary complication to underlying diagnoses (21), with common causes being identified as infections, malignancy, thrombotic conditions, neurosurgical procedures, or head trauma (1, 8, 21). In this cohort of patients presenting to the ICU, no identifiable cause was determined in many of the patients. In addition, many of those with primary CVST and without underlying diseases, presented with a longer duration of symptoms and a large number developed intracranial complications with ischemia or hemorrhage and death. Pediatric patients with both primary and secondary CVST develop morbidities with approximately 50% of these patients demonstrating changes in their functional scores at hospital discharge, consistent with existing literature (9, 22).

Secondary CVST in the weeks to months following traumatic brain injury is well described, particularly in the adult literature. In this cohort, 36% of secondary CVST diagnosed in the PICU were in children admitted with a primary diagnosis of acute traumatic or abusive brain injury. Interestingly, 45% of secondary CVST were diagnosed in children who had recently undergone a neurosurgical procedure. This population presents unique challenges in diagnoses specifically related to the limited neurologic examination available in many critically ill patients after acute brain injury or surgery requiring pharmacological paralysis and sedation. This study’s finding that greater than one-half of secondary CVSTs were diagnosed in children after they had a seizure presents an opportunity for possible earlier detection with the usage of continuous electroencephalography (cEEG). Guidelines for the management of pediatric severe traumatic brain injury recommend the continuous usage of EEG during the complete treatment course (23). Practice, likely due to resource constraints, does not align with the recommendation, with the median length of monitoring being 2 days in severe TBI (24). Prolonged cEEG monitoring in patients with acute brain injury or following brain surgery may detect changes before the child presents with clinical seizures. Finally, these findings support a consideration for vascular imaging in this subpopulation of critically ill patients, if they develop seizures during their admission, and the need for high index of suspicion for this diagnosis.

Using an area-based index of deprivation as an individual-level proxy in this study, children experiencing poverty and marginalization with regard to race or ethnicity may be at increased risk of developing both primary and secondary CVST. This finding does not appear to be driven by underlying anemia (possibly by way of food insecurity or health literacy), nor delays in presentation (by way of healthcare access disparities or health literacy) based on interrogation of our data. The proportion of marginalized children in this cohort does not reflect the distribution of children across the five quintiles in Ontario, where 36% of children are reported to reside in Quintiles 4 and 5 (25). This is the first time this association has been reported among patients with CVST and further work delving into malnutrition, infection, and diagnostic delays would be important.

The mortality observed in this cohort is higher than what has been previously reported in the literature (9, 13, 14). This difference may be attributed to the inclusion criteria of this study, which focused exclusively on critically ill children, whereas other studies typically report outcomes for all hospitalized patients with CVST. As a result, comparisons between this study and existing literature are inherently complex, given the difference inpatient populations. Another possible explanation for the higher mortality may be related to underlying patient characteristics that were not captured and which may be unique to the population served by this center.

This study has several limitations. First, it is a retrospective review conducted at a single center with a relatively small patient population, albeit in a rare disease in children. Although the detailed clinical descriptions of patient presentation, disease progression, and treatments administered offer valuable insights for clinicians, some of the therapies and findings may reflect institution-specific practices, thereby reducing the broader applicability of the results. Second, the analysis focuses on the prevalence of CVST differentiated by primary and secondary, over a limited timeframe within a single Canadian quaternary care center. As such, the findings may not accurately reflect trends in other geographic regions or over longer periods. Third, the limited number of cases restricted the ability to explore statistically significant associations between patient outcomes and clinical variables. And lastly, this cohort included patients admitted to pediatric critical care and therefore a representation of a more severe form of disease than what is encountered outside the ICU context.

Despite these limitations, the study has notable strengths. The narrow evaluation period minimizes variability in critical care practices, ensuring consistency in the supportive treatments delivered to patients. The described cohort also describes various types of CVST not previously described and identifies important differences in clinical history, risk factors, and hospital course. Finally, the detailed account of clinical features at presentation provides useful guidance for frontline healthcare providers working in emergency departments, urgent care settings, and community hospitals.

## CONCLUSIONS

This study describing children admitted to a PICU with cerebral venous sinus thromboses demonstrates that acute primary CVST is more common than secondary CVST, and both are associated with significant intracranial complications, functional morbidity, and mortality. In addition, this study reports a disproportionate number of children residing in neighborhoods with increased marginalization who were diagnosed with CVST. Future studies are required to better understand prognostic factors, the association between therapy and timing on functional outcomes, and the impact of marginalization on the risk of CVST.

## Supplementary Material
