# Supplementary material for: Cerebral Venous Sinus Thrombosis in Pediatric Critical Care
Source: Crit Care Explor. 2026 May 25;8(6):e1418. doi: 10.1097/CCE.0000000000001418 (PMC13200926; doi:10.1097/CCE.0000000000001418)
Supplement: Supplementary file 1 [file cc9-8-e1418-s001.pdf]

Supplemental Table 1. Patient Characteristics and Presenting Features

Characteristic	All (n=30)	Primary CVST (n=19)	Secondary CVST (n=11)
**ON-Marg Material Resources**[1]			
Quintile 1 (least marginalized)	0	0	0
Quintile 2	3 (11)	3 (17)	0
Quintile 3	6 (21)	4 (22)	2 (20)
Quintile 4	11 (39)	7 (39)	4 (40)
Quintile 5 (most marginalized)	8 (29)	4 (22)	4 (40)
**Baseline Functional Scores**			
FSS, median (IQR)	6 (6, 6.75)	6 (6, 6.5)	6 (6, 6.5)
Pediatric Overall Performance Category			
POPC 1	20 (67)	12 (63)	8 (73)
POPC 2	7 (23)	6 (32)	1 (9)
POPC 3	3 (10)	1 (5)	2 (18)
Pediatric Cerebral Performance Category			
PCPC 1	25 (83)	16 (84)	9 (82)
PCPC 2	3 (10)	3 (16)	0
PCPC 3	2 (7)	0	2 (18)
**Initial laboratory values**[2]			
Anemia at admission	18 (60)	13 (68)	5 (45)
Initial hemoglobin, median (IQR)	107 (88, 119)	102 (75, 115)	110 (90, 127)
Thrombocytopenia at admission	7 (23)	6 (32)	1 (9)
Initial platelet count, median (IQR)	270 (157, 404)	270 (102, 420)	264 (204, 331)

[1] Two of the 30 patients had missing ON-Marg Material Resource scores; one due to a missing postal code, and one due to census data suppression, therefore 18 were used as the denominator for Primary CVST and 10 was used for the denominator for Secondary CVST.

[2] Hemoglobin units: gr/dL. Platelet counts units: count * 10^9/L. Anemia was defined as hemoglobin <110 gr/dL and thrombocytopenia was defined at platelet count <150.

Abbreviations: ON-Marg: Ontario Marginalization Index, FSS: Functional Status Scale, POPC: Pediatric Overall Performance Category, PCPC: Pediatric Cerebral Performance, IQR: interquartile range
